# Bcl-2 expression in rituximab refractory cutaneous B-cell lymphoma

**DOI:** 10.1038/sj.bjc.6603762

**Published:** 2007-05-01

**Authors:** M Wobser, H Voigt, A O Eggert, R Houben, C S Kauczok, E B Bröcker, J C Becker

**Affiliations:** 1 Department of Dermatology, University of Wuerzburg, Josef-Schneider-Strasse 2, 97080 Wuerzburg, Germany

**Keywords:** cutaneous B-cell lymphoma (CBCL), rituximab, RKIP, bcl-2

## Abstract

Rituximab has been established as an effective and safe therapy for cutaneous B-cell lymphoma (CBCL). Different survival pathways, that is the Raf/MEK/Erk- or the p38MAPK cascade, have been suggested as downstream mediators of rituximab and may be involved in treatment failure. Biopsies from four patients, suffering from different subtypes of CBCL, which were obtained at various time points of relapse during or after therapy with 375 mg rituximab per m^2^ of body surface area, were analysed for the expression of CD20, CD3, Ki-67, Raf-kinase inhibitory protein (RKIP) and bcl-2 by immunohistochemistry. No CD20-loss variants, that is the suggested main tumour escape mechanism to rituximab therapy, were observed in any specimen of relapsing CBCL. Notably, the expression of proapoptotic RKIP remained increased in these tumour samples. This was concomitated by a constant to slightly reduced proliferation status as demonstrated by Ki-67 staining. However, relapsing CBCL exhibited a strong upregulation of the antiapoptotic molecule bcl-2 in comparison to pretherapeutic levels. The immunohistochemical analyses of this case series of rituximab refractory CBCL suggest that upregulation of bcl-2 may play a major role in therapy resistance.

Primary cutaneous B-cell lymphomas (CBCLs) generally show an indolent course. However, recurrences are observed in 25–68% of the patients (Servitje *et al*, 2002). For multilocular CBCL, systemic therapy with rituximab has evolved as an attractive therapeutic option.

The exact *in vivo* mechanisms of rituximab still remain elusive but likely include antibody-dependent cellular cytotoxicity (ADCC), complement-mediated lysis (CDC) and immune responses (Reff *et al*, 1994; Selenko *et al*, 2001). Furthermore, rituximab inhibits crucial survival pathways by upregulating the Raf-kinase inhibitory protein (RKIP), thus directly modifying the extracellular signal-regulated kinase1/2- and nuclear factor-*κ* B, respectively (Jazirehi and Bonavida, 2005).

Despite a high initial response rate, local or distant recurrence of CBCL is frequently observed in rituximab therapy. To delineate possible factors predicting or being associated with relapse, we scrutinised recurrent CBCL from four patients before, during and after systemic rituximab therapy.

## PATIENTS AND METHODS

### Patient characteristics

Four patients with multifocal CBCL (classified according to the WHO-EORTC classification for primary cutaneous lymphoma (Willemze *et al*, 2005)) were treated with rituximab intravenously at doses of 375 mg m^−2^ body surface area at the Department of Dermatology (University of Wuerzburg, Germany). For patient characteristics see Table 1. Before therapy and at different time points after initiation of therapy, skin biopsies were taken to confirm the diagnosis and to allow further work-up. Patients provided informed consent before any of these analyses.

### Immunohistochemistry

Tissue specimen from cutaneous sites of lymphoma were obtained by surgical excision, immediately fixed in formalin and embedded in paraffin. Deparaffinisation, antigen retrieval (s1699, Dako, Hamburg, Germany), endogenous peroxidase blocking and washing procedures were performed according to standard protocols. For staining, slides were incubated for 60 min with a murine anti-CD20 polyclonal antibody (Clone L26, Dako), a murine anti-CD3 polyclonal antibody (Clone 17A2, BD, Heidelberg, Germany), Ki-67 (Clone M7240, Dako), a rabbit anti-RKIP polyclonal antibody (Upstate Biotechnology, Lake Placid, NY, USA) or for 25 min with a murine anti-bcl-2 polyclonal antibody (Clone 124, Dako). Visualisation was performed with Multi Link Biotin Kit and Streptavidin HRP (K0690) and Vector Nova Red (Vector SK-4800, Linaris, Wertheim, Germany) according to the manufacturers' protocol. Evaluation was performed by two independent histologists.

## RESULTS

**Figure 1:**
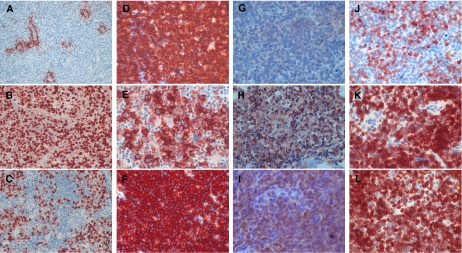
(**A**) CD3+ infiltrating T-lymphocytes surrounding the follicular CBCL lesion. Magnification × 10. (**B** and **C**) Proliferation status of the tumour cells as determined by ki-67 staining before (**B**) and during (**C**) therapy with rituximab. Magnification × 20. (**D**–**F**) Expression status of CD20 in relapsed CBCL before (**D**) and during (**E** and **F**) rituximab treatment. Magnification × 40. (**G**–**I**) RKIP expression at primary diagnosis (**G**) and during (**H** and **I**) therapy with rituximab. Magnification × 40. (**J**–**L**) Expression of antiapoptotic bcl-2 in recurring CBCL at diagnosis (**J**) and during rituximab application (**K** and **L**). Magnification × 40.

To this end, we analysed the presence of CD20 expression itself, as well as that of major intracellular downstream targets of rituximab by immunohistochemistry. We refrained from quantitative PCR analyses to avoid data confounding by the expression profile of inflammatory bystander cells in B-cell lymphoma infiltrates – which can be excluded by morphological analysis in immunohistochemistry. The quantitative evaluation of RKIP and bcl-2 expression was performed in tumour cell-enriched areas of CBCL, as determined by CD3 staining for detecting infiltrating T lymphocytes (Figure 1A).

At the time of first diagnosis, all tumours consisted of predominantly CD20-positive malignant lymphocytes (Table 2, Figure 1D). Recurrent disease demonstrated in nearly all patients a persistent CD20 expression (Figure 1F). A transient minor CD20 downregulation was observed in only one single biopsy (Table 2, Figure 1E).

The expression of proapoptotic RKIP in tissue specimen of patients before therapy with rituximab, was detectable only in few lymphoma cells (Table 2, Figure 1G). However, RKIP was remarkably upregulated in virtually all relapsed lymphoma specimen during or after therapy (Figure 1H and I). Indicative for proapoptotic and antiproliferative properties of upregulated RKIP, the proliferation status of B-cell lymphoma cells remained at least constant to slightly reduced under rituximab therapy (Figure 1B and C).

Bcl-2 expression was detected in all lymphoma specimen before therapy (Table 2), albeit only with a low to moderate intensity (Figure 1J). Notably, however, the expression of bcl-2 was consistently increased in relapsing disease (Figure 1K and L).

## DISCUSSION

The observed upregulation of proapoptotic RKIP *in situ* during rituximab therapy is likely to represent the direct intracellular signalling properties of rituximab (Vega *et al*, 2004; Jazirehi and Bonavida, 2005). RKIP belongs to the family of the metastasis suppressor genes, which inhibit cellular proliferation, growth and metastatic spread and induce apoptosis (Keller *et al*, 2005). Nevertheless, we observed rituximab refractory relapse in our patient cohort despite an overexpression of RKIP.

Different factors may contribute to this therapeutic failure of CD20-targeted therapies, including CD20 downregulation (Foran *et al*, 2001), altered signal transduction pathways (Pommier *et al*, 2004), circulating CD20 protein (Manshouri *et al*, 2003), polymorphisms in the Fc*γ*RIII receptor (Cartron *et al*, 2002) and an increase in complement-resistant proteins like CD55 or CD59 (Treon *et al*, 2001; Manches *et al*, 2003). The immunohistochemical analysis of the presented case series of relapsing CBCL lesions revealed an unchanged CD20 expression. In contrast, a highly increased bcl-2 expression in recurrent lesions was observed, thus providing an alternative explanation for treatment failure. The overexpression of proapoptotic factors, eg Mcl-1, bcl-2 or bcl-xL, has been implicated in cancer development, tumour progression and therapy resistance of B-cell lymphoproliferative diseases (Bannerji *et al*, 2003). The regulation of bcl-2 expression underlies a complex network of environmental stimuli, combined with altered signal transduction in lymphoma cells – which may abrogate the proapoptotic properties of rituximab and contribute to the observed therapeutic failure (Pommier *et al*, 2004). Interestingly, the relapsed CBCL in our patient cohort responded to other subsequent treatment modalities despite upregulation of bcl-2. Sensitisation to subsequent therapy may be conveyed by a counteracting downregulation of other antiapoptotic proteins by rituximab signalling; in this context, the observed increased expression of RKIP may be of importance, as it also functions as a negative regulator of antiapoptotic bcl-xL (Vega *et al*, 2004; Jazirehi and Bonavida, 2005). Hence, a complex network of interacting regulators of apoptosis is likely to be influenced by rituximab and the balance between pro- and antiapoptotic factors may determine the clinical outcome.

In summary, our results – albeit confined to a limited number of patients and the analysis of only two pro- or antiapoptotic signalling pathways, namely RKIP and bcl-2 – raise the hypothesis that the modulation of regulators of apoptosis may be important for the clinical effect of rituximab on CBCL. A more complex analysis of different signalling pathways, which includes potential cross-talks among apoptosis-regulating proteins in a larger patient cohort and parallel *in vitro* experiments, is warranted to establish the exact *modus operandi* of rituximab besides CDC and ADCC.

## Figures and Tables

**Table 1:** Patient characteristics of four patients exhibiting relapse under therapy with intravenous rituximab 375 mg m^−2^ of body surface area

Patients	4

*Gender*	
Male	1
Female	3

Age (years)	Mean: 67±6
	Range: 68–72

*Prior therapy*	
Excision	1
Radiotherapy	1
Oral antibiotics	1
Local steroids	1
Other	1
None	0

*Concomitant therapy*	
Excision	4
Radiotherapy	1
Chemotherapy	1
Other	0
None	0

*Subsequent therapy*	
Excision	3
Radiotherapy	2
Chemotherapy	1
Intralesional rituximab	1
Other	1
None	0

*Histology of primary diagnosis*	
FBCL	2
LBCL	2

*Best response to rituximab*	
CR	3
PR	1
SD	0
PD	0

Time to relapse (months)	Mean: 9±8
	Range: 0–24

*Best response to following therapy*	
CR	3
PR	0
SD	0
PD	0

Follow-up (years)	2±1

*Current status*	
Alive with disease	1
Alive without disease	3
Died of lymphoma	0
Died of unrelated cause	0

**Table 2:** CD20, RKIP and bcl-2 expression levels in specimen of cutaneous lymphoma relapse obtained at different time points since treatment start

		**CD20-positive cells (%)**					**RKIP-positive cells (%)**					**Bcl-2-positive cells (%)**				
**Patients**	**Timepoint of biopsy**	**<10**	**10–30**	**30–60**	**60–90**	**>90**	**<10**	**10–30**	**30–60**	**60–90**	**>90**	**<10**	**10–30**	**30–60**	**60–90**	**>90**
Patient 1	At diagnosis					x	x						x			
	At relapse after 4 months (during therapy)		x							x						x
	At relapse after 6 months (during therapy)				x					x						x
	At relapse after 12 months (during therapy)				x					x						x
Patient 2	At diagnosis		x				x							x		
	At relapse after 6 months (5 months since therapy cessation)		x							x				x		
	At relapse after 9 months (8 months since therapy cessation)		x							x					x	
Patient 3	At diagnosis				x				x							x
	At relapse after 13 months (6 months since therapy cessation)				x					x						x
Patient 4	At relapse after 4 months (2 months since therapy cessation)		x						x					x		
	At relapse after 30 months (28 months since therapy cessation)		x							x					x	

RKIP**=**Raf-kinase inhibitory protein.

Repetitive biopsies were obtained either during (patient 1) or after (patients 2–4) rituximab therapy.
